# Activating Transcription Factor 4 and X Box Binding Protein 1 of *Litopenaeus vannamei* Transcriptional Regulated White Spot Syndrome Virus Genes *Wsv023* and *Wsv083*

**DOI:** 10.1371/journal.pone.0062603

**Published:** 2013-04-24

**Authors:** Xiao-Yun Li, Li-Ran Pang, Yong-Gui Chen, Shao-Ping Weng, Hai-Tao Yue, Ze-Zhi Zhang, Yi-Hong Chen, Jian-Guo He

**Affiliations:** 1 MOE Key Laboratory of Aquatic Product Safety/State Key Laboratory for Biocontrol, School of Life Sciences, Sun Yat-sen University, Guangzhou, PR China; 2 School of Marine Sciences, Sun Yat-sen University, Guangzhou, PR China; Beijing Institute of Microbiology and Epidemiology, China

## Abstract

In response to endoplasmic reticulum (ER) stress, the signaling pathway termed unfolded protein response (UPR) is activated. To investigate the role of UPR in *Litopenaeus vannamei* immunity, the *activating transcription factor 4* (designated as *LvATF4*) which belonged to a branch of the UPR, the [protein kinase RNA (PKR)-like ER kinase, (PERK)]-[eukaryotic initiation factor 2 subunit alpha (eIF2α)] pathway, was identified and characterized. The full-length cDNA of *LvATF4* was 1972 bp long, with an open reading frame of 1299 bp long that encoded a 432 amino acid protein. *LvATF4* was highly expressed in gills, intestines and stomach. For the white spot syndrome virus (WSSV) challenge, *LvATF4* was upregulated in the gills after 3 hpi and increased by 1.9-fold (96 hpi) compared to the mock-treated group. The *LvATF4* knock-down by RNA interference resulted in a lower cumulative mortality of *L. vannamei* under WSSV infection. Reporter gene assays show that LvATF4 could upregulate the expression of the WSSV gene *wsv023* based on the activating transcription factor/cyclic adenosine 3′, 5′-monophosphate response element (ATF/CRE). Another transcription factor of *L. vannamei*, X box binding protein 1 (designated as LvXBP1), has a significant function in [inositol-requiring enzyme-1(IRE1) – (XBP1)] pathway. This transcription factor upregulated the expression of the WSSV gene *wsv083* based on the UPR element (UPRE). These results suggest that in *L. vannamei* UPR signaling pathway transcription factors are important for WSSV and might facilitate WSSV infection.

## Introduction

The endoplasmic reticulum (ER) is the primary subcellular organelle of eukaryotic cells. This organelle is a site of lipid synthesis, protein folding and protein maturation. The proteins locating in plasma membrane, Golgi apparatus and lysosomes, as well as the secreted proteins fold into their tertiary and quaternary structures in the ER [1]. Moreover, the ER is the major signal transducing organelle that recognizes and responds to homeostatic changes [2]. Conditions interfering with the function of the ER are collectively called ER-stress, which is induced by the accumulation of unfolded protein aggregates or by excessive protein traffic usually caused by viral infection [3].

The unfolded protein response (UPR) is the core pathway that eukaryotic cells use to cope with ER stress [4]. It is highly conserved and has been observed in eukaryotes. UPR contributes to cell survival by enhancing the protein folding capacity of the ER. Three pathways are involved in UPR, namely (1) [inositol-requiring enzyme-1 (IRE1)]-[X box binding protein 1 (XBP1)] pathway; (2) [double-stranded RNA-activated protein kinase (PKR)-like ER kinase (PERK)]-[Eukaryotic initiation factor 2α (eIF2α)]-[Activating transcription factor 4 (ATF4)] pathway, and (3) activating transcription factor 6 (ATF6) pathway [3], [4]. UPR decreases protein translation to restore normal cell functions, increases the production of molecular chaperones involved in protein folding, and activates the signaling pathways, resulting in the ubiquitination and subsequent degradation of misfolded proteins in the ER by proteasomes [5].

Under normal conditions, the immunoglobulin heavy chain binding protein (Bip) binds to the ER transmembrane protein sensors and inhibits their activation. Under ER-stress, Bip is occupied by the accumulated unfolded proteins, which frees the protein sensor [6]. In the PERK-eIF2α pathway, freed PERK spontaneously dimerizes and activates eIF2a S_(51)_ phosphorylation. Phosphorylated eIF2a has a reduced frequency of AUG initiation codon recognition. However, selective mRNAs, such as the mRNA of the transcriptional activator in PERK-eIF2a pathway, *ATF4*, are effectively translated and activated [7], [8]. In the IRE1-XBP1 pathway, the IRE1-Bip complex disintegrates, and IRE1 dimerizes. The IRE1 homodimers juxtapose the kinase domains for trans-autophosphorylation to stimulate the kinase and RNase activities that recognize the secondary structure of the *XBP1* mRNA and remove the consensus motifs in the hairpin loops [9]. Thus, resulting in a translational frameshift that generates a transcription factor containing a basic region leucine zipper (BRLZ) domain that can recognize an activating transcription factor/cyclic adenosine 3′, 5′-monophosphate response element (ATF/CRE) [10].

The Pacific white shrimp, *Litopenaeus vannamei,* is one of the most important penaeid shrimp in commercial fishing [11]. During culture, *L. vannamei* suffer from environmental stress, such as temperature shifts, sudden salinity changes, viral and bacterial infections, and heavy metal toxicity, which may induce ER-stress and harm the shrimp. Recently, the relationship between virus infection and the UPR in shrimp has become a constant concern, and researchers have found a complex relationship between them. For *Penaeus monodon*, heat shock protein70 (HSP70) responded to heat shock treatment, and knocked down *hsp70* results in the manifestation of severe white spot syndrome virus (WSSV) infections [12]. In the hepatopancreas and hemocytes of *Fenneropenaeus chinensis*, different *calreticulin* (*CRT*) expression profiles were observed upon WSSV challenge [13]. The *L. vannamei* IRE1-XBP1 pathway is activated under dithiothreitol (DTT) stimulation, heat shock treatment, or WSSV challenge [14]. Previous studies have shown that WSSV successfully usurps the immunity system of the host for its own gene regulation. For examples, the Janus kinase (JAK)-signal transducer and activator of transcription (STAT) signaling pathway is part of the antiviral response of arthropods, however, in *Penaeus monodon*, PmSTAT is activated in response to WSSV infection, and WSSV uses the PmSTAT as a transcription factor to enhance the expression of viral gene *ie1* [15]. Furthermore, two *L. vannamei* high-mobility group box proteins interact with transcription factors LvSTAT and LvDorsal to activate the promoter of WSSV *ie1* [16]. The Toll-like receptor mediated NF-κB pathways are essential for inducing immune-related gene expressions against bacterial, fungal and viral infections. The Toll receptor in *L. vannamei*, namely, LvToll1, LvToll2, LvToll3, and three putative Spätzle-like Toll ligands (LvSpz1-3) are upregulated after WSSV challenge [17]. The relationship between the WSSV genes and UPR transcription factors LvATF4 and LvXBP1 was investigated in this research. We demonstrated that in *L. vannamei* UPR was also activated by WSSV infection and LvATF4 and LvXBP1 upregulated expression of WSSV genes *wsv023* and *wsv083*, respectively.

## Materials and Methods

### 1. Cloning of LvATF4 from L. vannamei

Based on expression sequence tag (EST) sequences (available at www.marinegenomics.org, MGID428796), which are homologous to ATF4 of *Danio rerio, Glossina morsitans morsitans, Camponotus floridanus,* and some other species, specific primers were designed to obtain full length cDNAs. We obtained the 3′- and 5′-end cDNA sequences of *L. vannamei ATF4* (*LvATF4*) by rapid amplification of cDNA ends (RACE). The cDNA template for RACE-polymerase chain reaction (PCR) was prepared using the BD SMART RACE cDNA Amplification Kit (Clontech, Japan). The *LvATF4* 5′RACE1, *LvATF4* 3′RACE1 primers were used for the first round 5′-end and 3′-end RACE-PCR with a thermal cycler under the following conditions: denaturation at 94°C for 2 min, 7 cycles of 98°C for 10s, 68°C for 30s (decreasing by 1°C per cycle), and 68°C for 1.5 min, followed by 34 cycles of 98°C for 10s, 61°C for 30s, 68°C for 1.5 min, and final extension at 68°C for 5 min. The conditions for the second round 5′-end and 3′-end PCR using *LvATF4* 5′RACE2 and *LvATF4* 3′RACE2 primers were the same as the first round. The primer sequences are listed in Table 1. The PCR products were cloned into pMD-19 vector (TaKaRa, Japan) and subsequently sequenced (ABI PRISM, Applied Biosystems, USA).

**Table 1:** Summary of primers used in this study.

Primers	Sequence (5′-3′)
**For cDNA cloning**	
LvATF4-5RACE1	TTGATTGTCAGGCTGAGCAAGCTGGGTC
LvATF4-5RACE2	GAAGTTACAGCAGGGTTCATCAGGACATTA
LvATF4-3RACE1	ACGTTCCCCATCCAAAAGGAAGTCACGA
LvATF4-3RACE2	GGGTAGCGCCCTATCCAGAAAATAAACG
**For genes expression** a	
pACB-LvATF4-Kpn1-F	AAT**GGTACC**ATGGAGTACAACACGAACGGA
pACB-LvATF4-Apa1-R	A ATA**GGGCCC**TCTTTCTTTTTGAAAGCATCAGCT
pACB-LvXBP1u/s-F	TATGGTACCATGGCCAAGACGATCGTCATC
pACB-LvXBP1u-R	ATTGGGCCCGCCATCCCAGTTGGATTCCAG
pACB-LvXBP1s-R	ATTGGGCCCCGAAAGAGGGATGGGAACAATC
**For reporter genes** a,b	
pGL3B-wsv023-Kpn1-F	AAT**GGTACCC**TCACTAAAGAAAATAGTGTTTTTG
pGL3B-wsv023-Bgl2-R	TAT**AGATCT**GAGGGATGGTTGTTGATACTAC
pGL3B-wsv049-kpn1-F	AAT**GGTACC**CGTCTCCATACGATCCTTGTAG
pGL3B-wsv049-Hin3-R	TCG**AAGCTT**TGCCCAGAGATGGGAGTAACAC
pGL3B-wsv064-Kpn1-F	AAT**GGTACC**TTTCAAAGAATATGATATTGGGG
pGL3B-wsv064-Bgl2-R	TAT**AGATCT**TTCTTCAAGCACCTGACGGTAT
pGL3B-wsv069-Kpn1-F	AAT**GGTACC**AATATGATACCTGTTCTTCCGAT
pGL3B-wsv069-Bgl2-R	TAT**AGATCT**CAAAGAGATTTGTAGAGTCTTCAAAAT
pGL3B-wsv138-Kpn1-F	AAT**GGTACC**CACTCTTGTTCTTAATCAGACTAGATA
pGL3B-wsv138-Bgl2-R	TAT**AGATCT**CTAGTATGAGGTTAGTGTTTCGTCC
pGL3B-wsv242-Kpn1-F	AAT**GGTACC**AAGTCAACTAAGAGAAAAACACATC
pGL3B-wsv242-Bgl2-R	TAT**AGATCT**CCTTAAAAGCTGTTCCACCTTT
pGL3B-wsv256-Kpn1-F	AAT**GGTACC**TTTTTCAAACAATAAACTAACCACC
pGL3B-wsv256-Hin3-R	TCG**AAGCTT**GAAACCCAAGGAAGGGTTGC
pGL3B-wsv282-Sac1-F	TAT**GAGCTC**AGGTTGGTTGAGATAGAGAGTGAA
pGL3B-wsv282-Bgl2-R	TAT**AGATCT**ACCTCATTCTCCTCTTCAGATGA
PGL3B-wsv303-Kpn1-F	AAT**GGTACC**GGGAGAAGGTGGGTTTGAA
pGL3B-wsv303-Bgl2-R	TAT**AGATCT**TTGGAACAAGCACACCATGT
pGL3B-wsv306-Kpn1-F	AAT**GGTACC**ACGCCTTATGTTTTATTAGCGA
pGL3B-wsv306-Bgl2-R	TAT**AGATCT**GGAAGGCATTCTTAGACAAATC
pGL3B-wsv313-Kpn1-F	AAT**GGTACC**AAATCTTGAAGCATAGTTTTCTTC
pGL3B-wsv313-Bgl2-R	TAT**AGATCT**AACACTCCCGCACCTTCAGTTA
pGL3B-wsv321-Kpn1-F	AAT**GGTACC**TCGCATCTTCTTGTTGTGCC
pGL3B-wsv321-Bgl2-R	TAT**AGATCT**GCAATAACAAATGCAGCGATAA
pGL3B-wsv343-Kpn1-F	AAT**GGTACC**GCTGCTCTAGCCTGTTCCTTAA
pGL3B-wsv343-Bgl2-R	TAT**AGATCT**GGTTGAGCACATTTGCCTTAA
pGL3B-wsv406-Kpn1-F	AAT**GGTACC**AAACCTTTGATGACACAATTTT
pGL3B-wsv406-Bgl2-R	TAT**AGATCT**TCTCCTCCACCAAATACTGACA
pGL3B-wsv453-Kpn1-F	AAT**GGTACC**GAACCTCCACTTCCTCCGTC
pGL3B-wsv453-Hin3-R	TCG**AAGCTT**TGAAAAGGACAGAGCATCGG
pGL3B-wsv023dATF/CRE-F	ACTTTCTACGATCTTCTTCACGGA
pGL3B-wsv023mATF/CRE-F	ctcgaaggACTTTCTACGATCTTCTTCACGGA
pGL3B-wsv023ATF/CRE-R	GCAGATGTGGATTCTGTATCTGAA
pGL3B-wsv083-Kpn1-F	ATA**GGTACC**CTTGTAGCTTGAGTATTGGCAG
pGL3B-wsv083-Xho1-R	GAT**CTCGAG**GCCTATGGAAGAAACAACAAC
pGL3B-wsv083mATF/CRE-F	aactacacCCAGTGCAGTGTAGTGCCCAAC
pGL3B-wsv083ATF/CRE-R	ATGTAGAAGTAGTAGTGGTAAT
**For real-time RT-PCR**	
QPCR-LvATF4-F	GAAGTCTGGAGCTGGAGCATCA
QPCR-LvATF4-R	CAGGGACTCCAAAGGATGCTT
QPCR-LvEF1α-F	GCTGATTGCGCCGTACTCAT
QPCR-LvEF1α-R	TCACGGGTCTGTCCGTTCTT
**For RT-PCR**	
LvATF4-F	GGAGGACTACAGCAATACCA
LvATF4-R	AGGGCAGACTTGTCAATGGTA
**For dsRNA templates amplification**	
DsRNA-LvATF4-T7-F	GGATCCTAATACGACTCACTATAGGATGCTTGTCCCTCTGTCTG
DsRNA-LvATF4-R	ACAGGCGATGGAAGGGA
DsRNA-LvATF4-F	ATGCTTGTCCCTCTGTCTG
DsRNA-LvATF4-T7-R	GGATCCTAATACGACTCACTATAGGACAGGCGATGGAAGGGA
DsRNA-eGFP-T7-F	GGATCCTAATACGACTCACTATAGGCGACGTAAACGGCCACAAGTT
DsRNA-eGFP-R	ATGGGGGTGTTCTGCTGGTAG
DsRNA-eGFP-F	CGACGTAAACGGCCACAAGTT
DsRNA-eGFP-T7-R	GGATCCTAATACGACTCACTATAGGATGGGGGTGTTCTGCTGGTAG

a Nucleotides in bold indicate restriction sites introduced for cloning.

b Nucleotides in lower-case are the mutant sites.

### 2. Bioinformatics Analysis

The ATF4 protein sequences from other species in the database were searched and analyzed using the BLAST program (http://www.ncbi.nlm.nih.gov/BLAST/). Multiple sequence alignment was performed using the ClusterX v1.83 program. A neighbor-joining (NJ) phylogenic tree was constructed based on the deduced amino acid sequences of LvATF4 and other known ATF4 proteins using MEGA 4.0 software. Bootstrap sampling was carried out 1,000 times. Protein domains were predicted using the SMART program (http://smart.embl-heidelberg.de/).

### 3. WSSV Challenge and RNA Extractions for Real-time RT-PCR Analysis

The shrimp (approximately 6.0 g mean body weight) were sacrificed and their eyestalks, gills, heart, hepatopancreas, stomach, intestines, nerves, muscles, pyloric caecum, hemocytes, and epidermis were collected for tissue expression analysis. A WSSV challenge experiment was performed to investigate the *LvATF4* expression profile in the WSSV-infected shrimp. The WSSV challenge experiment method was described previously [18]. Moribund *L. vannamei*, which had white spots on the carapace and were confirmed to be WSSV-positive via one-step PCR, were used to prepare the inoculum for the challenge tests. The diseased shrimp muscle (2g) were homogenized in 20 mL of sterile 1× phosphate-buffered saline (PBS) (8 g NaCl, 0.2 g KCl, 1.44 g Na_2_HPO_4_, 0.24 g K_2_HPO_4_, pH adjusted to 7.4, dH_2_O added to 1 L), and then centrifuged at 5000×*g* for 15 min at 4°C. The supernatant was filtered through a 0.45-µm membrane and the sterilized supernatant was used for inoculation. Each healthy *L. vannamei* was injected intramuscularly at the second abdominal segment with 50 µL of WSSV inoculum (1×10^4^ virions) that was prepared from WSSV-infected shrimp following a published protocol [19]. Total RNA from the gills and hemocytes were isolated immediately and at 3, 6, 9, 12, 18, 24, 30, 36, 48, 72 and 96 h after injection. The total RNA samples were extracted and subsequently reverse transcribed into cDNA using PrimeScript RT Reagent Kit (TaKaRa). Real-time RT-PCR assays were performed as previously described. *LvEF1α* was used as the internal reference. The primer sequences are listed in Table 1.

### 4. Synthesis of Double-stranded RNA

The DNA templates of *LvATF4* dsRNA (designated as dsLvATF4) were prepared via PCR using the primer pairs, DsRNA-LvATF4-T7-F/DsRNA-LvATF4-R and DsRNA-LvATF4-F/DsRNA-LvATF4-T7-R (Table 1). The products with a T7 promoter were confirmed via sequencing. Subsequently, the products were used as templates for the sense and antisense RNA strands, and subjected to *in vitro* transcription using RiboMAXTM Large Scale RNA production System-T7 (Promega) following the protocol of the manufacturer. After the reaction, the DNA templates were incubated at 37°C with RNase-Free DNase (1 U/l g) for 15 min. The *in vitro* transcribed RNA products were then subjected to phenol/chloroform extraction, followed by isopropanol precipitation. An equal amount of sense and antisense RNAs were annealed to each other in 1× annealing buffer [20 mM potassium acetate, 6 mM Hepes-KOH (pH7.4), 6 mM MgOAc] at 90°C for 2 min to obtain dsRNA. The temperature was gradually decreased to 37°C, held for 1 h, and then placed at room temperature for another 1 h. The leftover single-stranded RNA template and the single-stranded overhang were degraded by incubating the annealing product with 0.1 µg of RNaseA at 37°C for 10 min followed by phenol/chloroform extraction and isopropanol precipitation. The *LvATF4* dsRNA was 442 bp in length. The templates of eGFP dsRNA (designated as dseGFP) were prepared via PCR using the primer pairs DsRNA-eGFP-T7-F/DsRNA-eGFP-R and DsRNA-eGFP-F/DsRNA-eGFP-T7-R (Table 1). The eGFP dsRNA was 504 bp in length.

### 5. Effect of Silencing LvATF4 upon a WSSV Challenge Test

The shrimp about 6.0 g body weight (50 shrimp/treatment obtained from the Zhuhai Shrimp Farm in Zhuhai, Guangdong Province, China) were intramuscularly injected with 6 µg (50 µL volume) of dsRNA or PBS. The shrimp were again injected after 48 h with either 50 µL of PBS (negative controls) or tissue homogenate at a 10^−3^ dilution (1×10^4^ virions, 50µL volume). Shrimp were kept in culture tanks for approximately 7 d following infection. Cumulative mortality was recorded every 12 h. For investigating the efficiency of RNA interference (RNAi) in this study, shrimp treated with dsLvATF4 or dseGFP were sacrificed at the third day after dsRNA injection [20], and the gills were collected for analyses. The gills of the untreated shrimp with dsRNA were also collected and used as a control. The total RNA extracted from the gills was reverse transcribed into cDNA using the PrimeScript RT Reagent Kit (TaKaRa) to serve as templates. Semi-RT-PCR assays were performed as described previously. *LvEF1α* was used as the internal reference. The primer sequences are listed in Table 1.

### 6. Dual-luciferase Reporter Assays

Expression vectors for the full length LvATF4, enhanced green fluorescent protein (eGFP), were constructed using pAc5.1/V5-His B (Invitrogen, USA). The PCR products were amplified using the primers pairs pACB-LvATF4-F/pACB-LvATF4-R and pACB-eGFP-F/pACB-eGFP-R (Table. 1), respectively. The PCR products were cleaved using the same restriction enzyme pairs and then ligated into the expression vector. The reporter genes, pGL3-wsv023, pGL3-wsv049, pGL3-wsv064, pGL3-wsv069, pGL3-wsv138, pGL3-wsv242, pGL3-wsv256, pGL3-wsv282, pGL3-wsv303, pGL3-wsv306, pGL3-wsv313, pGL3-wsv321, pGL3-wsv343, pGL3-wsv406 and pGL3-wsv453, were constructed by inserting the corresponding viral promoter regions into the pGL3-Basic, as described previously [16]. The pGL3-wsv023mATF/CRE and pGL3-wsv023dATF/CRE were constructed based on pGL3-wsv023 using the TaKaRa MutanBEST Kit (TaKaRa) according to the instructions of the manufacturer. Another set of reporter genes with a UPR Element (UPRE) motif that belong to the ATF/CRE family in WSSV genes promoters were constructed and scanned by the unspliced form LvXBP1 (LvXBP1u) and the spliced form LvXBP1 (LvXBP1s) (data not shown). The pGL3-wsv083mUPRE (primers are shown in Table 1) was constructed based on pGL3-wsv083 using the TaKaRa MutanBEST Kit (TaKaRa). *Drosophila Schneider 2* (S2) cells maintenance, DNA transfection, and reporter gene assays were performed as described previously [16].

## Results

### 1. Cloning of Full Length LvATF4 cDNA and Phylogenetic Analysis

**Figure 1:**
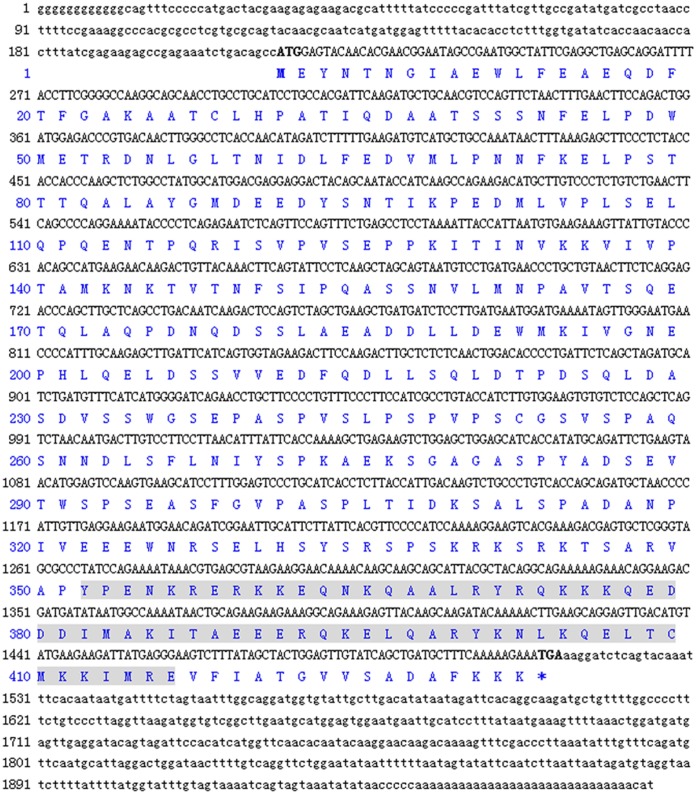
Nucleotide and deduced amino acid sequence of LvATF4. The ORF of the nucleotide sequences are shown in uppercase letters; the 5′- and 3′-UTRs are shown in lowercase letters. Nucleotides and amino acids are numbered on the left of the sequences. The conserved domains are shaded.

**Figure 2:**
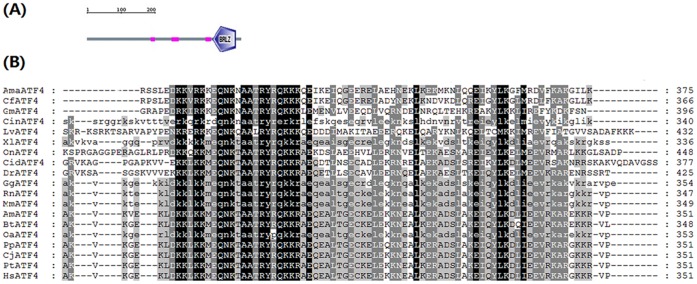
Multiple sequence alignment of the LvATF4 proteins. (A) Schematic representation of the structural domains of LvATFs. (B) The full names of the LvATF4 proteins and their corresponding sequence accession numbers are listed in the legend in Figure 3. The result shows a high homology of amino acid sequences at the conserved domains.

The full-length cDNA of *LvATF4* is 1972 bp long, includes a 213 bp 5′ untranslated region (UTR) and a 460 bp 3′-UTR with a poly (A) tail. The open reading frame (ORF) of *LvATF4* is 1299 bp long, and encodes a putative 433 amino acid protein (Fig. 1). Conserved domain analysis using the SMART program shows that LvATF4 contains a BRLZ domain (Fig. 2A),that is found in eukaryotic proteins with sequence-specific DNA binding activity [21].

**Figure 3:**
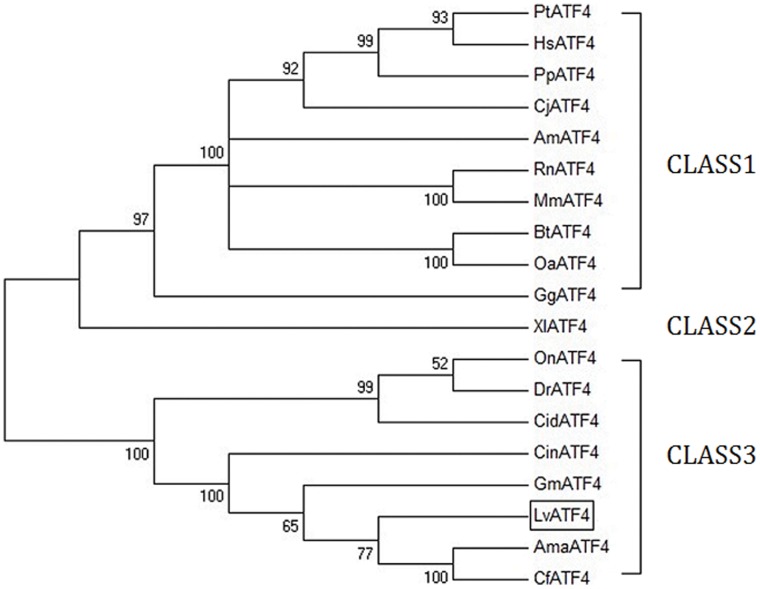
Phylogenetic analysis of the ATF4 proteins. Phylogenetic tree of the ATF4 proteins from invertebrates and vertebrates. The tree was constructed via a neighbor-joining algorithm using the Mega 4.0 program based on the multiple sequence alignment by ClusterX v1.83. The LvATF4 protein is marked with a box. CinATF4, *Ciona intestinalis* activating transcription factor 4 *(*GenBank accession no. BAE06319.1); AmaATF4, *Amblyomma maculatum* activating transcription factor 4 (GenBank accession no. AEO35749.1); CfATF4, *Camponotus floridanus* activating transcription factor 4 (GenBank accession no.EFN64595.1); GmATF4, *Glossina morsitans morsitans* activating transcription factor 4 (GenBank accession no.ADD20536.1); OnATF4, *Oreochromis niloticus* activating transcription factor 4 (GenBank accession no.XP_003443277.1); CidATF4, *Ctenopharyngodon idella* activating transcription factor 4 (GenBank accession no.AAS57931.1); DrATF4, *Danio rerio* activating transcription factor 4 (GenBank accession no.NP_001096662.1); XlATF4, *Xenopus laevis* activating transcription factor 4 (GenBank accession no.NP_001083212.1); GgATF4, *Gallus gallusactivating* transcription factor 4 (GenBank accession no.BAA76466.1); AmATF4, *Ailuropoda melanoleuca* activating transcription factor 4 (GenBank accession no.XP_002914604.1); BtATF4, *Bos taurus,* activating transcription factor 4 (GenBank accession no.NP_001029514.1); OaATF4, *Ovis aries* activating transcription factor 4 (GenBank accession no.ACJ15469.1); PpATF4, *Pan paniscus* activating transcription factor 4 (GenBank accession no.XP_003813296.1); CjATF4, *Callithrix jacchus* activating transcription factor 4 (GenBank accession no.XP_002763954.1); PtATF4, *Pan troglodytes* activating transcription factor 4 (GenBank accession no.NP_001239446.1); HsATF4, *Homo sapiens* activating transcription factor 4 (GenBank accession no.NP_877962.1); RnATF4 *Rattus norvegicus* activating transcription factor 4 (GenBank accession no.AAI58589.1); MmATF4, *Mus musculus* activating transcription factor 4 (GenBank accession no.AAH85169.1).

To investigate the relationship between LvATF4 and its homologues, multiple sequence alignment was performed (Fig. 2B) and phylogenetic trees were generated using the NJ method. These proteins were divided into three classes (Fig. 3). Class 1 contained the Mammalia and Aves ATF4 proteins, including GgATF4, AmATF4, BtATF4, OaATF4, PpATF4, CjATF4, PtATF4, HsATF4, RnATF4, MmATF4. Class 2 contained the ATF4 protein of amphibian,XlATF4. Class 3 contained fish, arthropod and tunicates ATF4 proteins, including OnATF4, CidATF4, and DrATF4, CinATF4, AmaATF4, CfATF4, GmATF4, and LvATF4. LvATF4 was most closely related to insect ATF4 proteins. The amino acid identity between AmaATF4 and LvATF4 was 47.4%. The ATF4 proteins showed a high homology with the amino acid sequence at the BRLZ domain (Fig. 2B).

### 2. LvATF4 Expression and Knockdown of LvATF4 in WSSV Challenged Shrimp

#### 2.1. LvATF4 Expression in WSSV Challenged Shrimp

**Figure 4:**
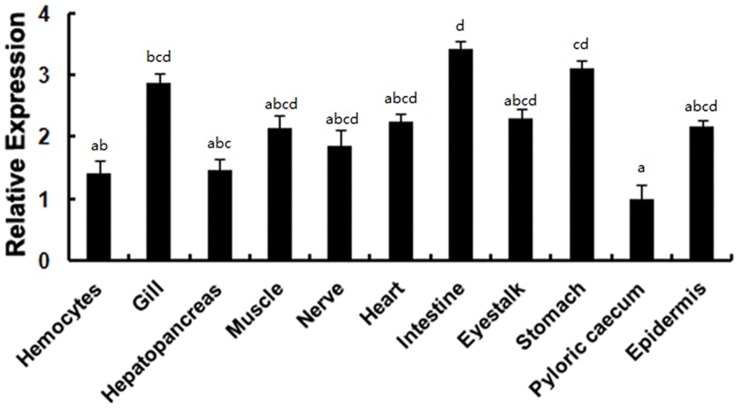
Expression profiles of *LvATF4* in tissues using real-time RT-PCR. Total RNA extracted from different tissues was reverse-transcribed into cDNA to serve as templates. The relative expression levels were normalized to *LvEF1α* and the expression of *LvATF4* in various tissues were compared against that in the gills. The results are based on three independent experiments and expressed as mean values ± S.D. Statistical significance was determined by one-way ANOVA. Bars with different letters indicate statistical differences (*p*<0.05).

**Figure 5:**
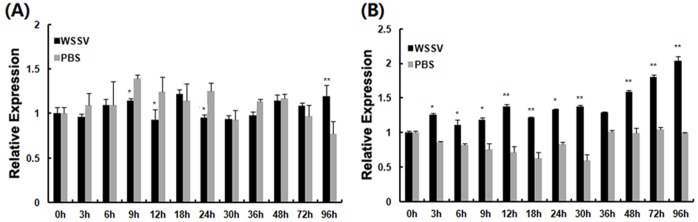
Expression of *LATF4* in hemocytes and gill of WSSV challeng shrimp. The mRNAs were collected at various time points (0, 3, 6, 9, 12, 18, 24, 30, 36, 48, 72, and 96 h) after injection. After the WSSV challenge or PBS injection at various times, the expression level of each gene was measured using real-time RT-PCR. The relative expression of *LvATF4* in hemocytes (A), in gills (B) were normalized with *LvEF1α* and compared against time zero. The bars represent the mean values ± S.D. of three replicates. The statistical significance was calculated using Student’s *t*-test (* indicates *p*<0.05 and ** indicates *p*<0.01 compared with control).

*LvATF4* expressions were detected in all the tissues examined. The expressions of *LvATF4* was statistical significanse different between each tissues. *LvATF4* was highly expressed in gills, intestines and stomach (Fig. 4). Considering that the LvATF4 with higher expression in the gills and hemocytes are important for shrimp immunity, the *LvATF4* in shrimp gills and hemocytes were investigated after WSSV challenge. The expression level of the gene at 0 h was used as the baseline, and the corresponding expression in the PBS group was used as the control. In hemocytes, the *LvATF4* expression was not significantly different between the WSSV-infected groups and mock-infected groups except 9, 12, 24, 96 hpi (Fig. 5A). Expression of LvATF4 was upregulated upon WSSV infection in gills after 3 hpi and increased by 1.9-fold (96 hpi) compared to the mock-treated group (Fig. 5B).

#### 2.2. Knockdown of LvATF4 reduced the cumulative mortality of WSSV infected shrimp

**Figure 6:**
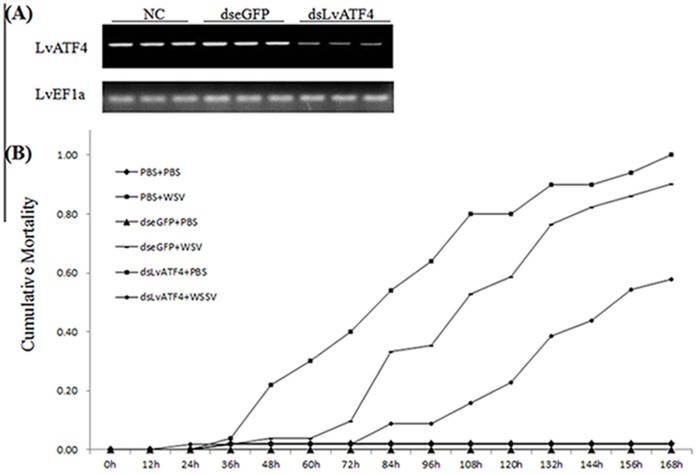
Shrimp cumulative mortality following treatment with dsRNAs and experimental infection with WSSV. (A) RT-PCR analysis of gene expression of *LvATF4,* the internal control was *LvEF1α*. Samples were taken 72 h after injection with indicated dsRNA; (B) Shrimp (n = 50) were injected intramuscularly with PBS, dsLvATF4 or dseGFP. 48 h after the initial injection, animals were injected with WSSV or PBS (negative control). Cumulative mortality was recorded every 12 h. Differences in cumulative mortality levels between treatments were analyzed by Kaplan–Meier log-rank χ2 tests.

Experiments were carried out to explore the effect of the downregulation of the *LvATF4* expression upon WSSV infection. The injection of dsLvATF4 reduced the expression of the *LvATF4* in the gills after 3 d, as shown in Fig. 6A. The injection of dseGFP did not induce the downregulation of the *LvATF4* expression in the gills. The shrimp treated with dsLvATF4 had a lower cumulative mortality after WSSV infection. The cumulative mortality was 8.77%, 15.79%, 38.60%, and 54.39% in the dsLvATF4 treatment group at 84, 108, 132, and 156 hpi, respectively. By contrast, the corresponding cumulative mortality in the control group was 54%, 80%, 90%, and 94%. The cumulative mortality was 100% after 168 hpi for the control group, whereas that of the dsLvATF4 treatment group was just 57.89% (Fig. 6B). The dseGFP injection failed to protect the shrimp from viral infection, similar to the control group (Fig. 6B).

### 3. Upregulation of WSSV Genes wsv023 by LvATF4 and wsv083 by LvXBP1s in S2 Cells

**Figure 7:**
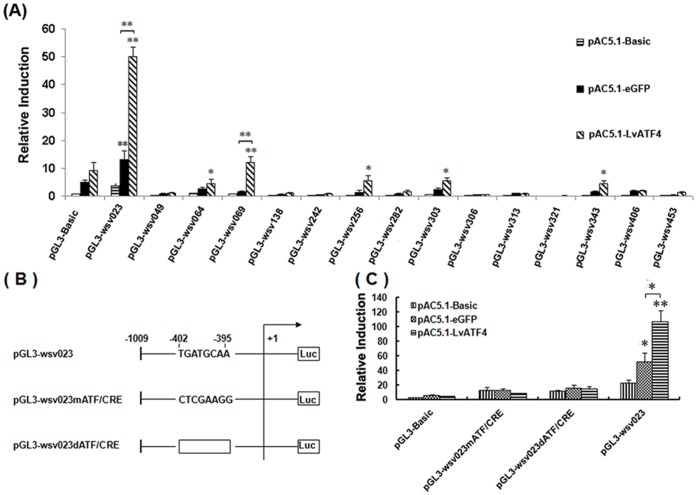
Activation of the *wsv023* promoters by LvATF4. (A) Screening of 15 WSSV gene promoters by LvATF4. (B) Schematic diagram of the *wsv023* promoter regions in the luciferase reporter gene constructs. For pGL3-wsv023mATF/CRE, the ATF/CRE in the *wsv023* promoters was replaced with (CTCGAAGG); and for pGL3-wsv023dATF/CRE, the ATF/CRE in the *wsv023* promoters was deleted (showed as square frame). +1 denotes the transcription initiation site for *wsv023*, and −1 indicates 1 bp before the translation initiation site. Luc denotes the firefly luciferase reporter gene. (C) Relative luciferase activity in S2 cells. The bars indicate mean values ± S.D. of the luciferase activity (n  = 3). The statistical significance was calculated using Student’s *t*-test (* indicates *p*<0.05 and ** indicates *p*<0.01 compared with control).

Based on the WSSV genome analysis, 15 genes (*wsv023, wsv049, wsv064, wsv069, wsv138, wsv242, wsv256, wsv282, wsv303, wsv306, wsv313, wsv321, wsv343, wsv406 and wsv453*) containing at least one putative ATF/CRE [TGACGT(G/C)A] within their promoter regions were selected. A luciferase reporter assay was performed to examine the relationship between transcription factors and these viral genes (Fig. 7A). Expression of pACB-LvATF4 significantly increased pGL3-wsv023 expression by approximately 10-fold (Fig. 7C). When the ATF/CRE element of *wsv023* promoter was mutated or deleted (Fig. 7B), the pGL3-wsv023 expression was significantly reduced by approximately 93% and 87% respectively comparing to the wild type (Fig. 7C). The expression of pGL-wsv069 was also activated by LvATF4, increased by approximately 6-fold (Fig. 7A).

**Figure 8:**
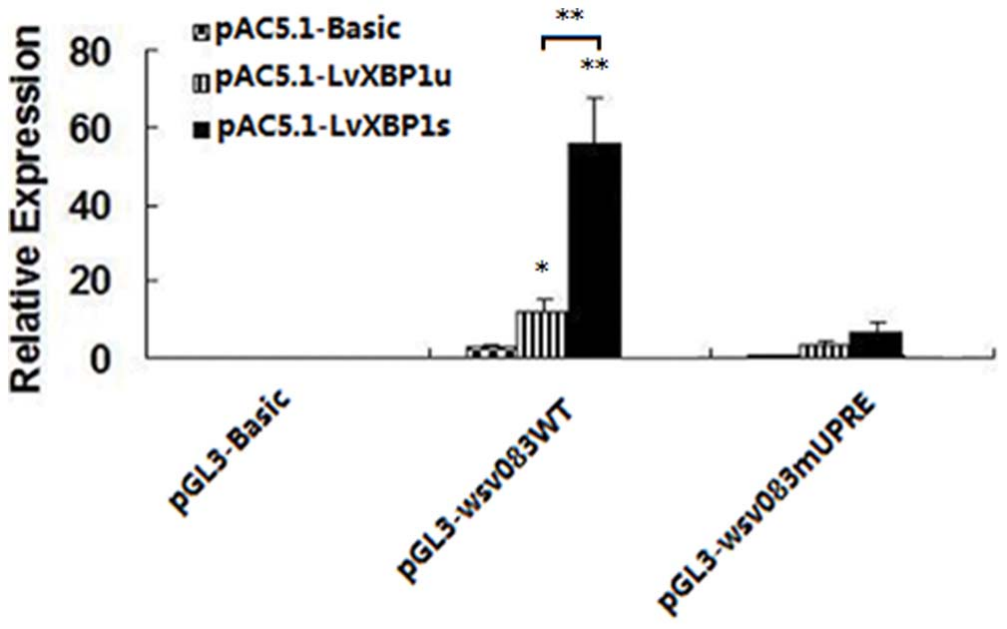
Activation of the *wsv083* promoter by LvXBP1s. Schematic diagram of the *wsv083* promoter regions in the luciferase reporter gene constructs. For pGL3-wsv083mUPRE, the UPRE in the *wsv083* promoters was replaced with (TGACGTGG). The results were based on three independent experiments and expressed as mean values ± SD. The statistical significance was calculated using Student’s *t*-test (* indicates *p*<0.05 and ** indicates *p*<0.01 compared with control).

It had been proved that WSSV infection leaded to LvXBP1 mRNA splicing, and the spliced form LvXBP1 mRNA (LvXBP1s)encoding polypeptide with ATF/CRE BRLZ domain [14]. As a potent transcription activator, XBP1 binds to the UPRE and to the ER stress-response elements I and II (ERSE-I and ERSE-II)in the promoter regions of the target genes [22]. Another set of reporter genes, containing at least one putative UPRE [TGACGTG(G/A)] within their promoter regions were selected. pGL3-wsv083 expression significantly increased by approximately 20-fold by LvXBP1s, whereas the expression of the *wsv083* promoter with UPRE element mutation was reduced 90% that compared with the wild type (Fig. 8).

## Discussion

Previous studies have demonstrated that the *L. vannamei* IRE1-XBP1 pathway is activated under WSSV challenge [14]. *LvXBP1* knocked down by RNAi results in a lower cumulative mortality of *L. vannamei* under WSSV infection. These results suggest that WSSV infection could cause ER stress and trigger UPR pathways in *L. vannamei*. Moreover, IRE1-XBP1 may be utilized to respond to the virus infection. In this study, LvATF4, a transcription factor of shrimp UPR, was identified and characterized. Under WSSV infection, the cumulative mortality of *L. vannamei* was lower in the dsLvATF4 treatment group than the control group. The reporter gene assays show that the transcription factor LvXBP1s upregulated the expression of the WSSV gene *wsv083* in the UPRE-dependent manner, and LvATF4 upregulated the expression of *wsv023* in an ATF/CRE-dependent manner. These results suggest that WSSV triggers ER stress and uses the transcription factor of the UPR pathway for its gene transcription regulation.

Several viruses hijack and stimulate the host immunity system to facilitate their life cycle. Cytomegalovirus (CMV) alpha [immediate early (IE)] genes could use NF-κB as a target for CMV enhancer activation [23], [24]. Human immunodeficiency virus type I, human herpes virus 8, and Epstein Barr virus have incorporated aspects of the NF-κB signaling into their life cycle and pathogenicity, and can thus induce NF-κB activation [25]. As the primary viral pathogen of shrimp, WSSV infection could activate the NF-κB pathway of the host. The NF-κB pathway in *L. vannamei* is activated by the WSSV protein wsv449 to facilitate *wsv303* and *wsv371* expressions [26]. NF-κB binds to the *wsv069* (*ie1*) promoter of WSSV and upregulates its activity [27]. Thus, WSSV could utilize the shrimp immunity system for survival, similar to other viruses.

The UPR regulates ER protein folding upon ER stress, and plays an important role in innate immunity [28]. Environmental stresses, such as temperature shifts, heavy metal toxicity and viral infection increase the levels of unfolded proteins in the ER lumen [29], [30], [31]. Viruses cause ER stress and consequently UPR activation by inducing host cells to produce large amounts of viral proteins, some of which undergo glycosylation and other modifications in the ER. For example, several members of the flaviviruses, including the West Nile virus, Japanese encephalitis virus, and dengue virus, activate the UPR pathway in a variety of mammalian cells [32], [33], [34], [35]. Viruses both induce and manipulate UPR to protect the host cells from an ER stress-mediated death, thereby permitting the translation of viral proteins and efficient viral replication [36]. UPR has emerged as a key target of host cells and viruses for controlling infection. Murine CMV (MCMV) regulates UPR in a manner similar to that of human CMV. The UPR modulatory ability is triggered by virion entry and enhanced by viral immediate-early and early gene expression. While initially vulnerable, MCMV becomes resistant to exogenous ER stress at the later stage of the infection [37]. In the Chinese shrimp, *F. chinensis,* WSSV challenge significantly enhanced the 78 kDA glucose-regulated protein (GRP78) expression, which also known as Bip, an important chaperon of UPR [38]. In the current study, *L. vannamei* UPR was also triggered by WSSV infection and WSSV can manipulate UPR to its advantage.

LvATF4 and LvXBP1 are the core transcription factors of *L. vannamei* URP. LvATF4 upregulated WSSV genes in an ATF/CRE-dependent manner. Among the 15 genes (*wsv023, wsv049, wsv064, wsv069, wsv138, wsv242, wsv256, wsv282, wsv303, wsv306, wsv313, wsv321, wsv343, wsv406 and wsv453*) with at least one putative ATF/CRE within their promoter regions *wsv023* was upregulated significantly. *Wsv023* is reportedly expressed at the early stage of WSSV infection, suggesting that it may be essential for WSSV [39], and its function still far from clear [40].This study might give some cues to uncover the function of *wsv023*, while further research was needed. *Wsv069* was also upregulated by LvATF4, but showed relatively smooth change comparing with that of *w023.* It had been reported that that WSSV protein IE1 function as transcriptional regulators and exhibit transactivation activity, DNA binding activity, and dimerization [41]. The relationship between LvATF4 and *wsv069* was needed further research. LvXBP1s with a putative BRLZ domain, significantly upregulated *wsv083*, which is an immediate early gene of WSSV and it was predicted to be a protein kinase 2 [39]. In a published work, wsv083 is a protein Ser/Thr kinase and inhibits the activity of focal adhesion kinase (FAK), involved in the processes of cell adhesion and spreading, which are crucial for invertebrate immune system [42]. The virus protein could inhibit cell adhesion and related kinase activity to evade the host immune response.

In summary, Our study demonstrated that the transcription factor LvATF4 and LvXBP1s could upregulate the expression of WSSV gene *wsv023* and w*sv083* respectively and the shrimp knock-down LvATF4 had a lower cumulative mortality after WSSV infection. WSSV utilizes the UPR to facilitate its infection by selectively using the UPR transcription factor to regulate its gene expression. Therefore, together with other immune pathways in *L. vannamei*, such as the JAK-STAT pathway and NF-κB pathway, UPR is an important pathway for WSSV infection and may be used to develop new strategies for controlling WSSV.
